# *De novo* RNA synthesis catalyzed by the Zika Virus RNA polymerase domain

**DOI:** 10.1038/s41598-017-03038-8

**Published:** 2017-06-02

**Authors:** Christina Calmels, Michel Ventura, Cindy Aknin, Mathieu Métifiot, Marie-Line Andreola

**Affiliations:** 1 Univ. Bordeaux, CNRS, MFP, UMR 5234, 146 rue Léo Saignat, Bordeaux cedex, 33076 France; 2 Fédération de Recherche “TransbioMed”, Bordeaux, France

## Abstract

Mosquito- and tick-borne pathogens including Chikungunya, Dengue, Japanese encephalitis, West Nile, Yellow fever and Zika virus, represent a new economic and public health challenge. In the absence of effective vaccines and specific therapies, only supportive regimens are administrated for most of these infections. Thus, the development of a targeted therapy is mandatory to stop the rapid progression of these pathogens and preoccupant associated burdens such as Guillain-Barre syndrome, microcephaly. For this, it is essential to develop biochemical tools to help study and target key viral enzymes involved in replication such as helicase complexes, methyl-transferases and RNA-dependent RNA polymerases. Here, we show that a highly purified ZIKV polymerase domain is active *in vitro*. Importantly, we show that this isolated domain is capable of *de novo* synthesis of the viral genome and efficient elongation without terminal nucleotide transferase activity. Altogether, this isolated polymerase domain will be a precious tool to screen and optimize specific nucleoside and non-nucleoside inhibitors to fight against Zika infections.

## Introduction

Zika virus (ZIKV) is a mosquito-borne member of the genus *flavivirus* within the *Flaviviridae* family. While ZIKV infections in humans are mainly asymptomatic (in 80% of cases), a smaller proportion of patients present benign febrile symptoms also called Zika fever. Guillain-Barre syndrome (GBS) is characterized by a paralysis of the peripheral nervous system and usually affects 1 to 2 out of 100,000 persons per year. Yet in countries where Zika infection is present, an unexpected increase of GBS has been observed. The prevalence of these neurological reversible damages is about 20-fold higher in Zika infected patients. Thus, Zika infection appears to be an trigger of GBS^1–3^. In addition, the increase occurrence in fetal microcephaly has been linked to the recent Zika virus outbreak as ZIKV was detected in the brain tissue of deceased foetus^4, 5^. Accordingly, Zika has been declared a public health emergency of national importance in Brazil^6^. Because there isn’t a vaccine nor a treatment specifically developed to block the worldwide expansion of this epidemic, there is an urgent need to develop a targeted therapeutic strategy which will require a better understanding of the virus replication.

The viral genome is a (+)-sense single-stranded RNA of approximately 10,800 nucleotides containing a single open reading frame (ORF). The Cap-dependent translation of the full-length polyprotein is followed by a maturation step leading to the generation of three structural proteins [capsid (C), precursor membrane (prM), and envelope (E)], and seven non-structural proteins (NS1, NS2A, NS2B, NS3, NS4A, NS4B, and NS5). The non-structural proteins associate themselves into a replication complex within membranous structures at/or derived from the endoplasmic reticulum. Efficient replication of the viral genome relies on the presence of two regulatory elements flanking this large ORF, the untranslated regions (the 5′ UTR and 3′ UTR). Actually, the RNA-dependent RNA polymerization is catalyzed by the NS5 protein which also harbors a N-terminal methyl-transferase domain (MTase) which activity combined to NS3’s 5′RNA phosphatase activity enables the capping of the nascent genomic RNA. Because the synthesis of an intermediate negative strand and subsequently new genomic copies is obviously essential, the RNA-dependent RNA polymerase (RdRp) activity constitutes a target of choice for drug development.

As of today, research on Zika has been focusing on developing diagnostic tests, setting of working cellular models of ZIKV and bio-physic approaches on certain viral proteins such as the NS2B-NS3 helicase complex^7^ or the MTase domain of NS5^8^. That being said, little biochemical studies has been published on these essential non-structural proteins while it is clearly needed for a better understanding of the molecular process of infection and pathogenicity^9^. Only recently Hercik *et al*. described the purification of a ZIKV full-length NS5 protein (MTase and RdRp domains)^10^. In this study, the full-length NS5 was capable of an uncharacterized nucleotide incorporation using a primed template enabling the authors to test nucleoside analog inhibitors. Here, we report the purification of the NS5 isolated RdRp domain and characterized its enzymatic activity. Notably, we found that the RdRp domain is active in the absence of the MTase domain and is capable of *de novo* synthesis that was not limited to terminal nucleotide transferase activity using both homopolymers and specific RNA templates mimicking the natural viral UTR substrate. Of note, since the original submission of this work, several new studies have been published on ZIKV RdRp purification and inhibition that corroborates our results^11–14^.

## Results

### ZIKV RdRp domain delineation, protein expression and purification

**Figure 1:**
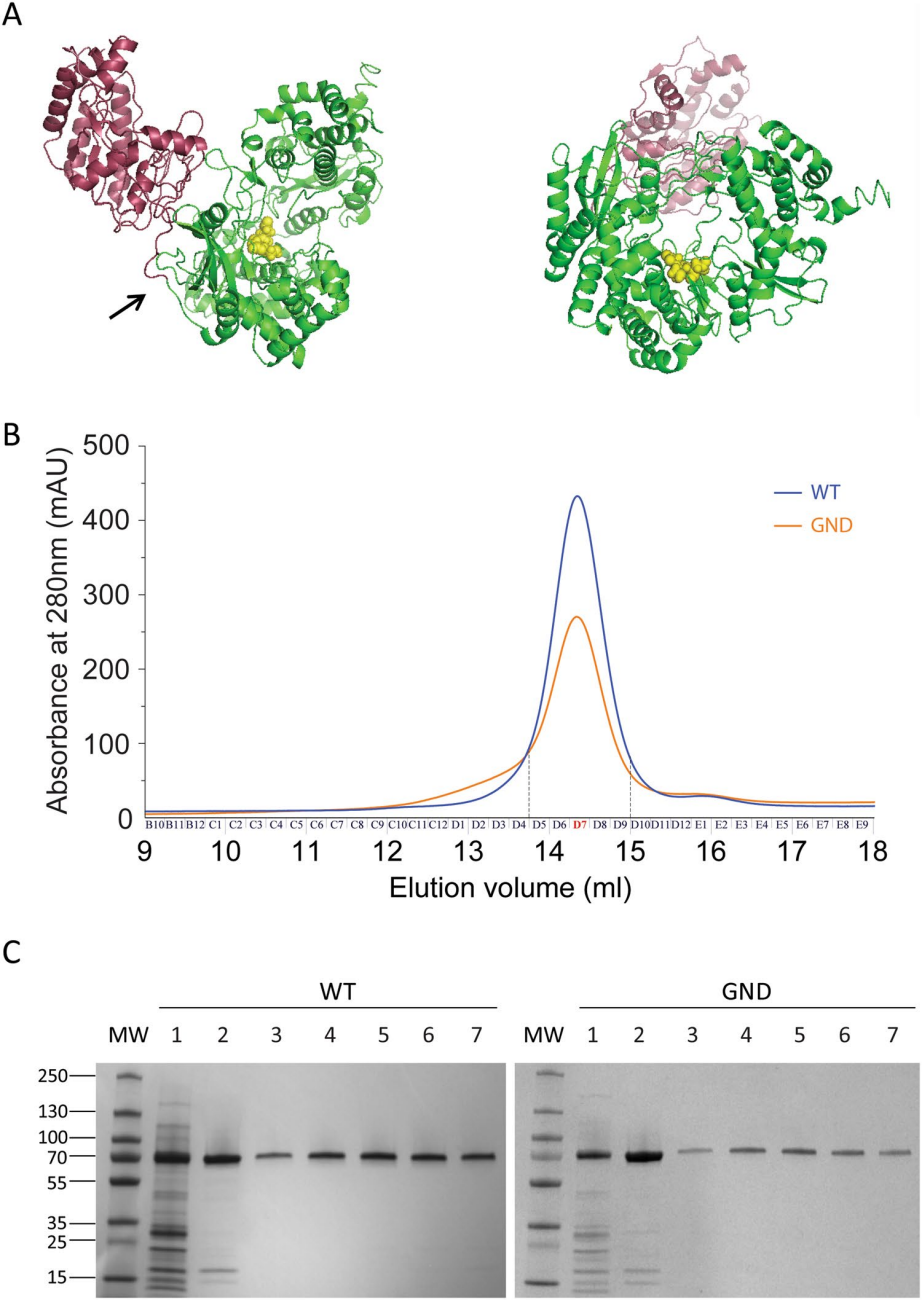
Structure prediction of NS5 and purification of ZIKV polymerase. (**A**) Structure prediction of the NS5 protein of ZIKV. The putative polymerase domain is in green and the MTase domain in purple. The arrow shows the limit between the MTase and the RdRp domain that was cloned. The three amino acids of the potential active site GDD appear as yellow spheres. Images were obtained following a 45° rotation. (**B**) Size exclusion chromatography profiles. Chromatograms correspond to the retention profile of the WT enzyme (blue) and catalytic mutant GND (orange). (**C**) SDS-PAGE analysis of polypeptides throughout the purification of WT (left) and catalytic mutant GND (right) ZIKV RdRp. MW corresponds to the molecular weight. Lane 1 corresponds to 4 µg of total protein pooled from the nickel column elution. Lane 2 corresponds to 3 µg of total protein pooled from the heparin column. Lanes 3 to 7 correspond to 0.4 µg of protein from fractions D5 to D9 obtained during size exclusion Proteins were stained with Coomassie Brillant blue R250.

Our aim was to study the isolated RdRp domain of NS5. Based on previous studies on the MTase domain of West Nile virus^15^, we used the cleavage site sequence GLVKRR/GG described between NS4 and NS5 to define the ZIKV NS5 N-terminus. Then, the 903 amino acid long protein from the SPH2015 strain^16^ was used to perform a 3D structure prediction using the I-TASSER bioinformatics web tool^17, 18^. The model identifies 2 distinct domains connected by a short linker (Fig. 1A). The first domain comprising amino acid residues 1–274 constitutes the MTase domain (in purple). The second domain from residue 275 to 903 adopted a typical right hand structure that was expected as the signature for a polymerase domain (Fig. 1A, in green).

The nucleic acid sequence corresponding to residues 275 to 903 (629 amino acids) from the SPH2015 strain was optimized for bacterial expression to avoid the use of rare codons, a sequence coding for an 8xHis tag was added in 5′ and silent mutations were introduced in the body of the gene to eliminate the BamH*I* cleavage site for cloning purposes (see Material and Methods). The resulting 1914 nucleotides long DNA was obtained by gene synthesis and cloned in a pET-21a vector. After expression in Rosetta cells, the RdRp isolated domain was purified using a regular Nickel affinity column, followed by a heparin column and subsequently a gel filtration column. To ascertain that the observed activity can be attributed to the purified NS5 RdRp and not a potential remaining contaminant, we designed a catalytically inactive version by mutating the active site from _664_GDD_666_ to _664_GND_666_. Mutation was introduced in the polymerase sequence and the mutant protein purified through the same procedure that was developed for the WT enzyme. The elution profile of both enzymes after exclusion chromatography was similar and showed a unique pic (Fig. 1B). Following separation on SDS-PAGE and Coomassie Brillant blue staining, purity of both proteins appeared to be close to homogeneity (Fig. 1C, >98%). The fraction D7 was used for subsequent enzymatic activity assays for both the WT and mutant enzymes.

### ZIKV RdRp isolated domain is active *in vitro*

**Figure 2:**
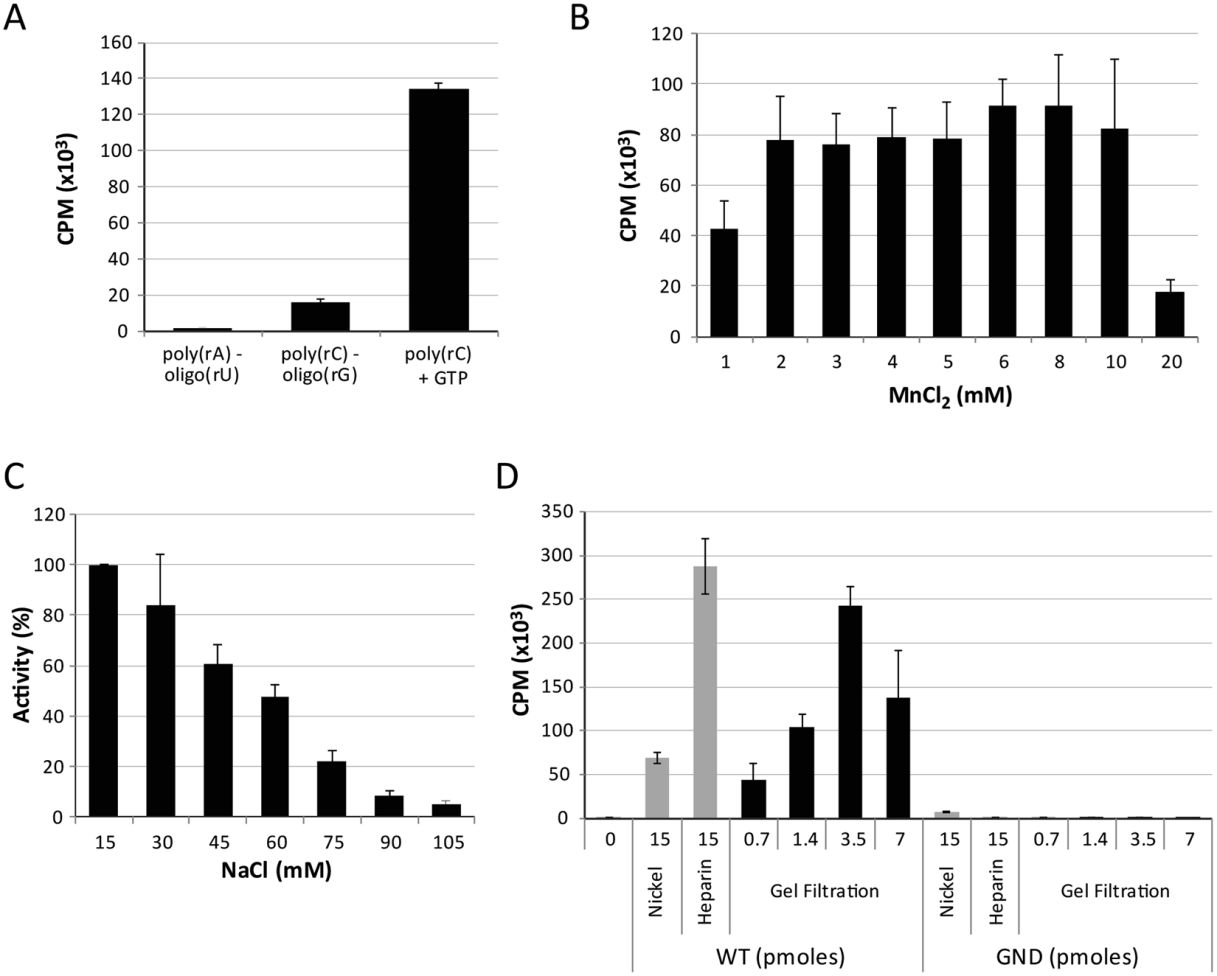
ZIKV RdRp enzymatic activities using homopolymeric templates. (**A**) Template utilization by ZIKV RdRp. Radionucleotide incorporation was monitored using 3.5 pmoles of WT enzyme (fraction D7 from gel filtration, see Fig. 1B) and various template-primer combinations. (**B**) Effect of the metal cofactor on nucleotide incorporation. (**C**) Impact of increasing concentration of NaCl on radionucleotide incorporation. The assay was conducted in conditions similar to panel A using the poly(rC) substrate and increasing concentrations of MnCl_2_ and NaCl, respectively. Because the RdRp was purified in a buffer containing NaCl, the lowest salt concentration reachable is 15 mM (no salt added). (**D**) *De novo* RNA synthesis activity of ZIKV RdRp throughout the purification stages. Pooled fractions eluted from the nickel column and from the heparin column were tested at 15 pmoles of total proteins for the WT and GND mutant polymerases. In parallel, fraction D7, corresponding to the main retention pic after gel filtration was assayed at 0.7, 1.4, 3.5 and 7 pmoles. Means and standard deviations derives from three independent determinations.

RNA synthesis was first assayed using Mn^2+^ as metal cofactor with various non-specific template-primer combinations (Fig. 2). The highest activity was obtained with a poly(rC)-oligo(rG) substrate which led to approximately a 10-fold higher incorporation rate of ^3^H-labeled nucleotides than the poly(rA)-oligo(rU) substrate (Fig. 2A). The incorporation of ^3^H-labeled nucleotides was again 10-fold more efficient when a poly(rC) substrate was used without primer (from about 15,000 cpm to more than 135,000 cpm, Fig. 2A). To optimize this ribonucleotide incorporation activity, several parameters were tested. Varying the concentration of Mn^2+^ had no drastic effect between 2 and 10 mM (Fig. 2B). Increasing concentrations of NaCl (from 15 mM brought with the enzyme to 105 mM) decreased the reaction efficiency (Fig. 2C). Thus, subsequent reaction conditions were set to 3 mM MnCl_2_ and 15 mM NaCl.

To further assess the gain in purity of the RdRp domain along our purification procedure, we used an aliquot of each purification steps in this *de novo* RNA synthesis assay and compared the WT activity to the activity of a presumed catalytically inactive GND mutant (Fig. 2D). Using 15 pmoles of proteins from the nickel column pool led to good ribonucleotide incorporation with the detection of more than 69,000 cpm. In the same condition, the fraction obtained with the GND mutant still exhibited a basal activity with around 7,500 cpm, probably resulting from co-purified contaminants. Additional purification with a heparin column greatly reduced the activity observed in the GND mutant fractions and only close to background signal was observed (600 cpm). In parallel, the activity of the pooled fractions containing the WT enzyme was increased by about 5-fold with 15 pmoles of proteins leading to almost 300,000 cpm. Finally, gel filtration appeared to be an important step to further purify the enzyme as almost the same level of incorporation (>250,000 cpm) was reached with only 3.5 pmoles of RdRp, corresponding to a 4-fold increase in activity (Fig. 2D). When tested, the GND ZIKV RdRp mutant was expectedly unable of *de novo* activity on incorporation using the poly(rC) template. Altogether, it appeared that all three steps included in our purification procedure were required in order to obtain an active RdRp exempt from other polymerase contaminant.

### ZIKV RdRp isolated domain efficiently catalyzed polymerization without terminal nucleotide transferase activity

**Figure 3:**
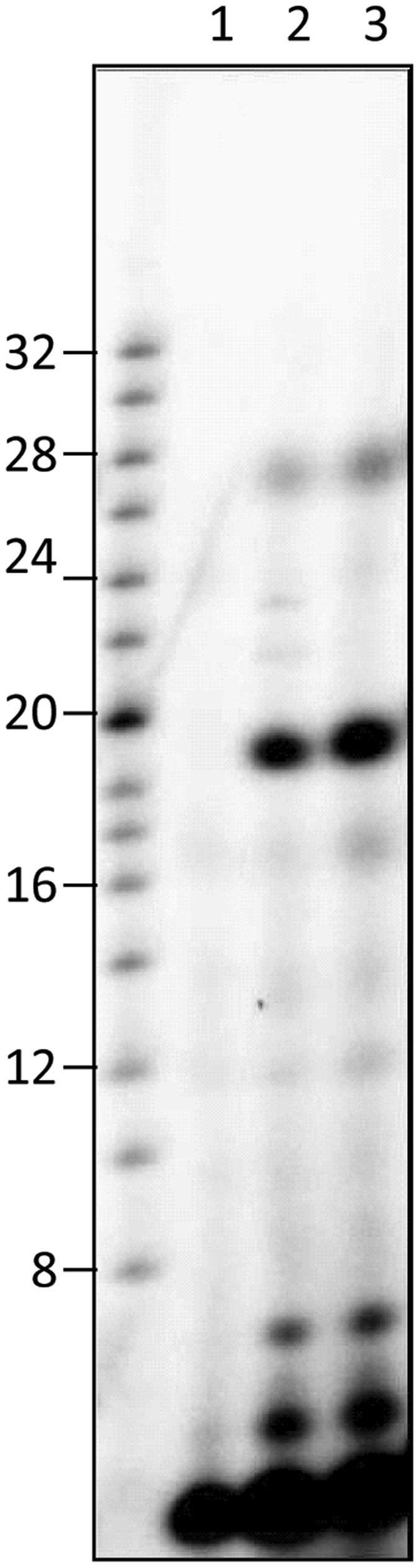
ZIKV RdRp enzymatic activity using oligo(rC) as template. *De novo* RNA synthesis was performed on oligo(rC) template in the presence of [α-^32^P]GTP. Reaction products were separated on 12% polyacrylamide denaturing gels (7 M urea). Autoradiography was performed using an imaging plate (Fujifilm) and images were obtained with a FLA-5000 Imaging System (Fujifilm). Markers from Amersham ranging from 8–32 bases were 5′-end labeled and loaded as control on the left of the gel. Lane 1, reaction mix without enzyme. Lanes 2 and 3 correspond to the reaction performed with oligo(rC) or oligo(rC) blocked with cordycepin as template, respectively.

Because simple incorporation of ribonucleotide in a filter-based precipitation assay does not discriminate the nature of incorporation (terminal transferase *vs de novo* synthesis leading to the replication of a template), we performed similar assays in the presence of [α-^32^P]-rGTP and visualized the products on a polyacrylamide gel (Fig. 3). For this purpose, an oligoribonucleotide containing 20 consecutive cytidines [oligo(rC)] was used as template. The main product observed migrated at the size of the substrate *i*.*e*. 20 nucleotides (Fig. 3, lane 2). Moreover, additional bands appeared specifically in the presence of the RdRp: products of lower apparent size (faster migration at the bottom of the gel) that could correspond to abortive elongations and longer products (slower migration above the migration level of the 20-mer molecular weight). Because there were abortive elongation events, it appeared that the isolated RdRp was capable of *de novo* initiation and elongation. However, we cannot rule out whether the 20-mer product was solely obtained from synthesis without at least in part terminal transferase activity. Such activity would lead to a product similar in length that might not be discriminated on a gel. Thus, we performed the same assay using a substrate which 3′ end has been blocked by cordycepin. In this context, a similar profile was observed with the main product migrating as the 20-mer marker (Fig. 3, lane 3). Thus, the activity observed in this assay was indeed solely from a polymerase activity and not a terminal transferase activity. Still, products with an apparent higher molecular weight were observed even with the substrate blocked with cordycepin. Because the substrate used is based on an oligo(rC) sequence, the product formed was an oligo(rG). Accordingly, it is possible that this G-rich product adopted non-canonical secondary structures such as G quartets which would affect their migration.

### The isolated RdRp domain specifically replicated ZIKV natural templates

**Figure 4:**
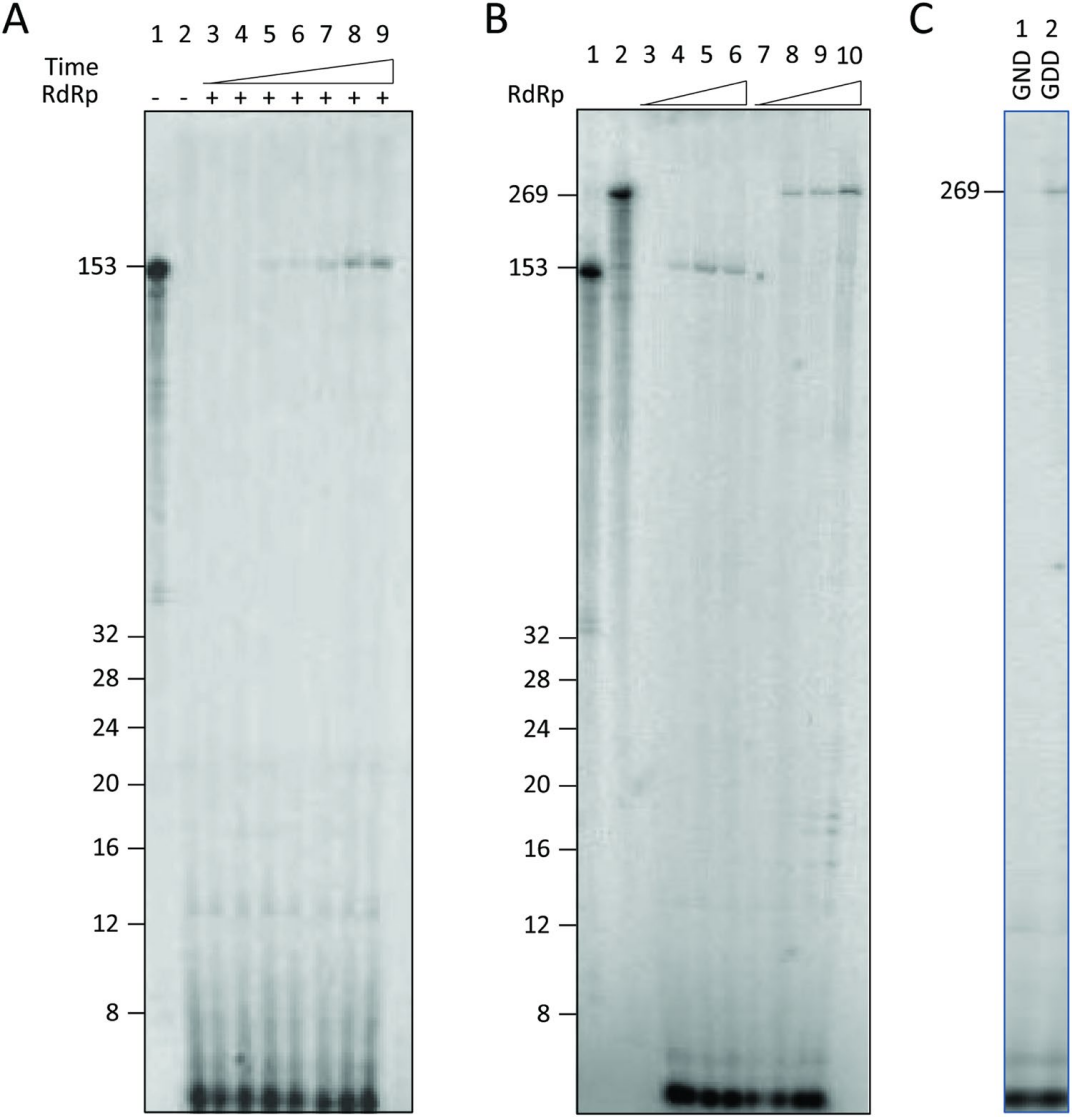
ZIKV RdRp enzymatic activities using natural templates. RNA substrates of 153 nucleotides and 269 nucleotides corresponding to the 3′-end of the (−) strand were obtained by *in vitro* transcription in the presence of [α^32^P]-UTP and subsequently used as molecular weight markers. Corresponding unlabeled RNAs were used as template to assay for the *de novo* RNA synthesis of the ZIKV RdRp as described in Material and Methods. (**A**) Time course experiment monitoring the ZIKV RdRp enzymatic activity (1.4 pmoles) using a 153–nucleotide long RNA as template. Lane 1: radiolabeled 153 nucleotides template. Lane 2: no enzyme. Lanes 3 to 9: reaction was pursued for 0, 5, 10, 15, 30, 60, 180 min, respectively. (**B**) Impact of the substrate length on WT RdRp activity. Lane 1 and 2: radiolabeled templates corresponding to 153 and 269 nucleotides, respectively. Lanes 3 to 6: synthesis using the 153-nucleotide long RNA substrate. Lanes 7 to 10: synthesis using the 269 nucleotides RNA substrate. Respectively 0, 0.7, 1.4, 3.5 pmoles of RdRp were used. (**C**) Lanes 1 and 2: Respectively, 1.4 pmoles of the GND mutant and WT RdRp (GDD) were used with the 269-nucleotide long RNA as template. Reactions were carried out for 2 h and products were separated on 12% polyacrylamide denaturing gels (7 M urea). Autoradiography was performed using an imaging plate (Fujifilm) and images were obtained with a FLA-5000 Imaging System (Fujifilm).

Then, the ability of the ZIKV RdRp to replicate *in vitro* its natural template was explored. RNA synthesis activity was assayed using 153 nucleotides of the 3′-end of the (−) strand. This sequence corresponds to the entire UTR that the RdRp is expected to recognize to synthesize the genomic (+) strand. During a time-course experiment, we observed the accumulation of a product at the expected size (Fig. 4A). Accordingly, it appears that the ZIKV RdRp is proficient for the synthesis of full-length products with a plateau reached after about 1 hour of reaction. Production of these full-length products was also dose-dependent as the quantity of product increased with the concentration of protein used in the assay (Fig. 4B, lanes 4–6). This indicated that purified ZIKV RdRp can efficiently use *in vitro* the 3′-end of the (−) strand RNA as template without any primer.

Second the RNA synthesis was assayed using a longer RNA as template. Actually, unpublished *in vitro* data obtained in our laboratory indicated that adding portion of the genomic ORF to the UTR sequence increased the replication efficiency of the RdRp from hepatitis C virus (HCV NS5B), compared to using the UTR alone as template. To investigate the possibility that additional sequences/structures may improve the RNA synthesis by the ZIKV polymerase, we generated a new RNA template of 269 nucleotides and compared the activity of the RdRp on this substrate to that of the 153 nucleotide-long template described above (Fig. 4B). In both cases, full-length products were observed (lanes 4–6 and lanes 8–10 for the 153 and 269 nucleotide-long RNAs, respectively). Even if the reaction efficiency appeared similar, additional bands were observed only for the 269 template. These stop products occurred after incorporation of 18 to 20 nucleotides as estimated on gel and attested a lack of processivity specific of this matrix. This suggested that secondary structures may be formed in this longer template that seemed to be absent or less stable in the shorter 153 nucleotide-long substrate, explaining the synthesis blockade.

Finally, to ascertain that the activity was due to the RdRp activity, the GND mutant was assayed using a natural 269 RNA as template. As expected, no synthesis was detected in conditions where the 269 product was observed using the WT enzyme (Fig. 4C, lanes 1 and 2).

## Discussion

Here, we describe the purification of an active ZIKV RdRP isolated domain. The enzyme is fully proficient for *de novo* RNA synthesis and elongation without terminal transferase activity. In addition to homopolymers, it successfully replicated full-length product on its natural RNA template [generating the (+) strand 5′ UTR from the negative template]. Yet, this activity was limited to the use of Mn^2+^ as a metal cofactor and Mg^2+^ dependent activity was observed only with an oligo(rC) substrate. In agreement with our results, a *de novo* activity was also described in a concomitant study by Xu and collaborators using a ZIKV minigenome RNA^12^. In addition, a primer dependent activity was also recently described using a homopolymeric template^12^ or a short template/primer system corresponding to the 5′ end of the genome as primer and the 3′ end of the antigenome as template^11^. In parallel, Hercik and collaborators described the purification of the ZIKV NS5 with both the MTase domain and RdRp domain leading to a Mg-dependent activity^10^. However, the substrate used was different [a poly(rU) template with an oligo(rA) primer] and bypassed the *de novo* initiation expected for *flavivirus* polymerases. Besides, the experimental setting used did not enable to distinguish if the active incorporation observed was indeed synthesis or a terminal transferase activity. Importantly, recent structural studies in the context of other flavivirus NS5 proteins have shown that the MTase domain may affect the RdRp overall activity thanks to allosteric interactions. Thus, it is tedious to rule out the impact of the metal cofactor in ZIKV NS5 polymerase activity. In past decades, a tremendous effort has been made to develop efficient and specific viral polymerase inhibitors. While first generation drugs such as AZT presented important drawbacks with serious side effects, rigorous drug development has led to the identification of more advanced and selective drugs. Our work demonstrates that the isolated ZIKV RdRp domain can be used *in vitro* to evaluate specific compounds and will help the repositioning of already approved drugs able to inhibit other viral polymerases. A potential lead could be molecules developed against the HCV NS5B protein such as sofosbuvir and other 2′-C-methylated nucleosides that have recently been shown to be active against ZIKV NS5 *in vitro* or the ZIKV replication in Vero cell^10, 14, 19, 20^. However, *in silico* studies performed on the ZIKV polymerase have predicted that most HCV NS5B inhibitors could be active against ZIKV^21^. Yet, among 30 nucleotides analogs tested in another study using cellular models, only few molecules were able to inhibit Zika infection^22^. This discrepancy highlights the need to biochemically validate the activity and mechanism of action of these inhibitors on the RdRp activity. In addition, this will enable the screening of chemical libraries which will also enable the identification of new drugs that similarly to HIV-1 non-nucleoside RT inhibitors may target more specifically the ZIKV polymerase preventing deleterious side effects.

## Experimental Procedures

### Expression vector construction

The coding sequence of ZIKV RdRp domain used in this study corresponds to the SPH2015 strain that has been optimized for codon usage using the GENEius software (Eurofins Genomics GmbH) followed by Pro codon optimization for bacterial expression (Genbank No. KY364193). The adapted gene harbored an internal BamH*I* recognition site that was subsequently removed and the sequence corresponding to an 8xHis tag was added in 5′ of the sequence. The corresponding DNA coding for a 72 kDa protein was obtained by gene synthesis (Eurofins Genomics GmbH, Munich) and inserted in the multi-cloning site of the pET-21a vector between BamH*I* and Xho*I* sites.

### Expression and purification of ZIKV NS5 RdRp domain

Rosetta cells were transformed with the pET-21a-ZIKVRdRp and bacteria are grown on LB plates containing ampicillin (100 µg/ml) and Chloramphenicol (34 µg/ml) overnight at 37 °C. Selected clones were expanded in 500 ml of liquid medium with the same antibiotics until an OD of 0.8 is reached (37 °C with agitation at 220 rpm). Protein expression was then induced by addition of IPTG at a final concentration of 2 mM and culture overnight at 18 °C and 220 rpm. After centrifugation at 2500 rpm for 5 min, bacteria pellet were resuspended with 20 ml of lysis buffer [50 mM Tris-HCl pH 8.0, 150 mM NaCl, 10 mM imidazole, 0.001% reduced Ttriton, 10% glycerol, 1.5 mg/ml lysozyme, 0.02 mg/ml DNase I (Roche), 1 mM phenylmethylsulfonyl fluoride (PMSF), and proteases inhibitors (Roche)] and incubated for 10 min at room temperature. After 3 × 20 sec sonication, soluble proteins were clarified by centrifugation 30 min at 12000 rpm (RC-5B, SS-34 rotor). The supernatant was applied onto a Nickel crude Hitrap column (1 ml, GE Healthcare) equilibrated with buffer A (50 mM Tris-HCl pH 8.0, 150 mM NaCl, 10 mM imidazole, 0.001% reduced Triton, 10% glycerol). After washing of the column with 5 volumes of buffer A, bound proteins were eluted in one step using 250 mM imidazole. Fractions containing the RdRp domain were determined by SDS-PAGE, pooled and diluted 1.5 fold with buffer B (corresponding to buffer A without NaCl and imidazole) before loading onto a Heparin-HiTrap column (1 ml, GE Healthcare) equilibrated with buffer B. Proteins elution was performed following a one-step increase in NaCl to 250 mM. Fractions containing the RdRp domain were determined by SDS-PAGE, pooled (1.2 ml) and concentrated by centrifugation on Amicon 30 kDa until a final volume of about 200 µl for optimal loading onto a Superdex 200 10/300 column (24 ml). Size exclusion was performed with a running buffer composed of 50 mM Tris-HCl pH 8.0, 150 mM NaCl, 0.001% reduced Triton and 10% glycerol. Purity of the protein was evaluated by SDS-PAGE. Concentration was measured using a NanoDrop 2000 spectrophotometer (Thermoscientific).

### Site-directed mutagenesis

Site-directed mutagenesis was performed on the pET21a-ZIKRdRp plasmid (7323 bp) following a standard PCR-based protocol using degenerated primers obtained from MWG Biotech. The amplification reaction was performed using a step at 98 °C for 30 seconds, followed by 20 cycles of 10 seconds at 98 °C, followed by 45 seconds at 60 °C and 1 minute per kb at 72 °C; and finally a step of 10 minutes at 72 °C. Amplification products were subjected to *Dpn*I digestion for 1 hour at 37 °C (10 U/50 µl reaction). DH5α chemically competent cells were transformed with the digestion product. After plasmid extraction (standard miniprep from Macherey-Nagel following manufacturer’s instructions), the presence of the desired mutations and the integrity of the ZIKV RdRp sequence were verified by DNA sequencing (MWG Eurofins). Primers sequences for mutagenesis were ATGGCAGTCTCAGGCAATGATTGTGTGGTGAAACC TATCGATGACC and CACAATCATTGCCTGAGACTGCCATGCGTTTCAAG CGGTCCCACCC. Primers AGGAGGTGTTAGAAATGCAA and TTATCCCAGCCAGTGGATGG were used for forward and reverse gene sequencing respectively.

### *In vitro* transcription for substrate preparation

The plasmid pEx-5UTRms3UTR was constructed to contain the 5′UTR and 3′UTR from the SPH2015 strain separated by a multi-cloning site with the following sequence GGATCCAAGCTTCTCGAGGCGGCCGCTCTAG. RNA sequences corresponding to 3′-end of the (−) strand were chosen to be used as substrate in the polymerase activity assay. PCR was performed using the Phusion Hotstart Flex DNA Polymerase (Biolabs) with primers designed to introduce a T7 RNA polymerase promoter enabling the generation of substrate RNAs by *in vitro* transcription. Primers sequences were GTTGTTACTGTTGCTGACTCAGAC along with TAATACGACTCACTATAGGGCATATTGACAATCCGGAATCCTCC or TAATACGACTCACTATAGGGCTCAAAAAGGCTAGAATTGCCAAG to produce DNA templates used to generate the 153 and 269 nucleotides long RNAs, respectively. After dsDNAs purification using phenol/chloroform extraction and precipitation, substrate RNAs were obtained by *in vitro* transcription using the MEGAscript kit (Ambion) according to manufacturer’s instructions. After DNase I digestion (TurboDNase, 2 U for 15 min at 37 °C) acidic phenol/chloroform extraction and precipitation, purity and integrity of the generated RNAs were determined using capillary electrophoresis on a Bioanalyzer 2100 (Agilent).

### Enzymatic activities

Reactions were performed for 2 hours at 30 °C with various amounts of purified RdRp in a reaction buffer containing 20 mM Tris-HCl pH 8.0, 1 mM DTT and 13.2 U RNasin (Promega). For primer-dependent reactions, 0.48 OD of poly(rA)-oligo(rU) or poly(rC)-oligo(rG) were incubated with 2 µCi [^3^H]-XTP (rUTP at 12.2 Ci/mmole and rGTP at 41.8 Ci/mmole) and 3 mM MnCl_2_. For *de novo* synthesis, catalysis was performed in the presence of (i) a poly(rC) template (0.4 OD) and 200 µM rGTP, (ii) 1 µM oligo(rC) (20-mer with a 3′-OH extremity or blocked with cordycepin) and 5 µCi [α^32^P]-GTP (3000 Ci/mmole), (iii) 1.5 pmoles of RNA templates, 0.5 mM rATP, 0.5 mM rCTP, 5 µCi [α^32^P]-GTP (3000 Ci/mmole) and 5 µCi [α^32^P]-UTP (3000 Ci/mmol). *De novo* reactions were performed in the presence of 3 mM MnCl_2_ except for oligo(rC) where MgCl_2_ (5 mM) was used.

For filter-based precipitable assays, reactions (20 µL) were stopped by addition of 1 ml cold 10% trichloracetic acid (TCA) plus 0.1 M sodium pyrophosphate. The precipitate was filtered through nitrocellulose membrane, washed with 2% TCA, dried and the radioactivity determined using a Packard Liquid Scintillant Analyser 2100TR with Perkin Elmer scintillation mixture.

For gel-based assays, reactions were stopped by addition of 7 M urea. Products were purified by addition of phenol/chloroforme/isoamylalcool (25:24:1, v:v:v) and subsequent nucleic acids precipitation. After washing and drying of the pellet, products were resuspended in loading buffer [formamide containing 1% sodium dodecyl sulfate (SDS), 0.25% bromophenol blue, and xylene cyanol]. Products were separated on 12% polyacrylamide denaturing sequencing gels. Autoradiography was performed using an imaging plate (Fujifilm) and images were obtained with a FLA-5000 Imaging System (Fujifilm).
